# Role of PMR4 and PDLP1 in priming of early acting penetration defense by resistance-inducing β-amino acids

**DOI:** 10.1016/j.isci.2024.109299

**Published:** 2024-02-20

**Authors:** Chia-Nan Tao, Jurriaan Ton

**Affiliations:** 1 School of Biosciences, Institute for Sustainable Food, The University of Sheffield, Sheffield S10 2TN, UK

**Keywords:** Plant biology, Interaction of plants with organisms, Molecular plant pathology

## Abstract

R-β-homoserine (RBH) and β-aminobutyric acid (BABA) induce resistance against the oomycete *Hyaloperonospora arabidopsidis* (*Hpa*) in Arabidopsis, which is based on priming of multiple defense layers, including early acting penetration resistance at the cell wall. Here, we have examined the molecular basis of RBH- and BABA-primed defense by cell wall papillae against *Hpa*. Three-dimensional reconstruction of *Hpa*-induced papillae by confocal microscopy revealed no structural differences between control-, RBH-, and BABA-treated plants after *Hpa* challenge. However, mutations affecting POWDERY MILDEW RESISTANCE 4 or PLASMODESMATA LOCATED PROTEINs (PDLPs) only impaired BABA-induced penetration resistance and not RBH-induced penetration resistance. Furthermore, *PDLP1* over-expression mimicked primed penetration resistance, while the intensity of GFP-tagged PDLP1 at germinating *Hpa* conidiospores was increased in BABA-primed plants but not RBH-primed plants. Our study reveals new regulatory layers of immune priming by β-amino acids and supports the notion that penetration resistance is a multifaceted defense layer that can be achieved through seperate pathways.

## Highlights

• The β-amino acids RBH and BABA prime cell wall defenses in Arabidopsis
• BABA-primed cell wall defense relies on PDLPs and PMR4
• BABA and RBH prime cell wall defense via different pathways and mechanisms

## Graphical abstract

**Figure:**
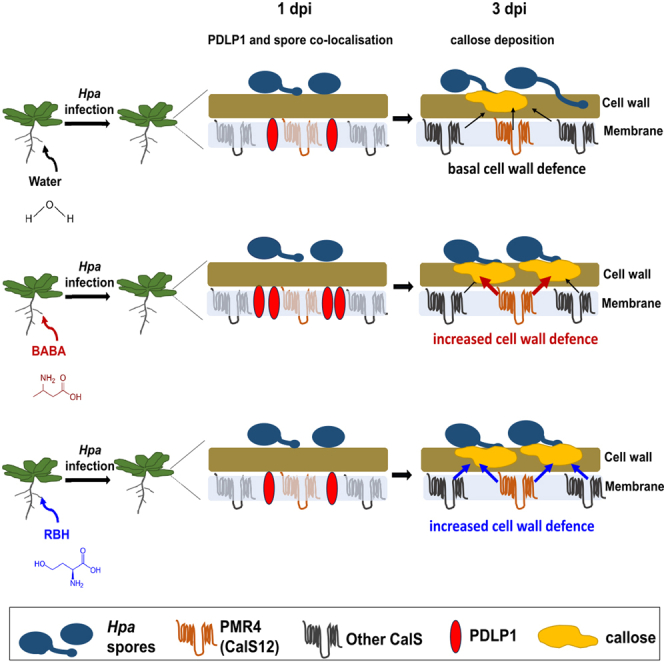

## Introduction

Although plants lack specialized immune cells to acquire immunity, they can develop an increased defensive capacity after exposure to pests or diseases, which protects against future assaults. This so-called induced resistance (IR) is typically long-lasting and mediated by immune priming, which mediates a faster and/or stronger immune response against future challenges by pests or diseases.^1^^,^^2^ Apart from biological elicitors, such as pathogens, herbivores, and rhizosphere-colonizing microbes, selected chemicals can trigger IR as well, including damage- and microbe-associated molecular patterns and plant-endogenous stress signaling compounds, such as salicylic acid (SA), jasmonic acid (JA), and β-aminobutyric acid (BABA). Among these resistance-inducing chemicals, BABA offers protection against an exceptionally wide spectrum of environmental stresses across a range of taxonomically unrelated plant species.^3^ BABA is produced by plants under conditions of (a)biotic stress,^4^ and perceived by the aspartyl-tRNA synthetase impaired in BABA-induced disease immunity 1 (IBI1), which controls downstream SA-dependent and -independent defense pathways of BABA-IR.^5^^,^^6^^,^^7^ Although BABA-IR is effective against numerous stresses, it can be phytotoxic at higher doses, causing significant reductions in plant growth.^5^^,^^8^ This undesirable effect is caused by the inhibitory binding of the active R-enantiomer of BABA to the L-aspartic acid-binding pocket of IBI1,^6^ leading to uncharged tRNA^Asp^ accumulation and general control nonderepressible 2 (GCN2)-dependent inhibition of gene translation.^5^

To identify less phytotoxic analogs of BABA, a previous study screened structurally related β-amino acids for induced resistance to *Hpa* without concurrent plant stress,^6^ resulting in the identification of R-β-homoserine (RBH). This β-amino acid induces resistance in Arabidopsis against biotrophic and necrotrophic pathogens without growth penalty.^6^ Interestingly, despite the structural similarity between RBH and BABA, the underlying IR pathways differ. Even though RBH and BABA use the same transporter to enter the cells (lysine histidine transporter 1; LHT1),^9^ RBH is not perceived by the IBI1 receptor.^6^ Furthermore, unlike BABA, RBH primes jasmonic acid- and ethylene-dependent defenses against the necrotrophic fungus *Plecosphaerella cucumerina.*^6^ Remarkably, however, both RBH and BABA prime the deposition of callose papillae, which arrest early colonization by the biotrophic Oomycete *Hyaloperonospora arabidopsidis* (*Hpa*).^6^ In other plant-Oomycete interactions, BABA has been reported to prime phenotypically similar cell wall defences.^3^^,^^10^ However, little is known about the pathways controlling this chemical priming of penetration defense.

Callose is a (1,3)-β-polyglucan in cell walls with numerous physiological functions, including pollen development, regulation of plasmodesmata permeability, and plant defense.^11^ Twelve callose synthase (*CalS*) genes have been identified in the Arabidopsis genome,^12^ of which *CalS12,* also known as powdery mildew resistant 4 (*PMR4*)*,* is the dominant CalS for pathogen-induced callose.^13^^,^^14^ PMR4-dependent callose is induced by damage- and microbe-associated molecular patterns at relatively early stages of plant-biotic interactions.^11^^,^^14^^,^^15^^,^^16^ Callose is thought to improve plant defense via different mechanisms; apart from reinforcing the primary cell wall to mechanically resist apoplastic pathogen colonization and cellular parasitization, it forms a matrix in which antimicrobial compounds can be deposited.^15^^,^^17^ The coordination of callose deposition involves regulation by plasmodesmata-located proteins (PDLPs), which contain a conserved transmembrane helix domain and two extracellular DUF26 domains that show similarity to carbohydrate-binding proteins.^18^ PDLPs have been found to control plasmodesmatal callose deposition and permeability.^19^^,^^20^^,^^21^ Moreover, PDLP1 co-localizes with *Hpa* haustoria to mediate PMR4-dependent callose deposition during the relatively advanced stages of the interaction.^22^ However, whether these proteins play a role in callose-associated penetration resistance at earlier stages of the interaction remains unknown.

Here, we have investigated the role of defense-related callose and PDLPs in priming of early acting penetration defense by RBH and BABA. Although IR by these agents involves multiple defense layers, IR from both chemicals is associated with primed deposition of callose-rich papillae during the early stages of infection by *Hpa.*^6^ However, the regulatory mechanisms underpinning this primed penetration defense against *Hpa* remain largely unknown. Using a combination of microscopy approaches and genetic resources, we show that RBH- and BABA-induced penetration resistance differ in their requirement of PMR4, despite similar structural morphologies of the *Hpa*-arresting callose papillae. We furthermore show that BABA primes the early co-localization of plasmodesmata located protein 1 with *Hpa* conidiospores to mediate induced penetration resistance.

## Results

### Morphology of primed callose papillae arresting early *Hpa* colonization

**Figure 1:**
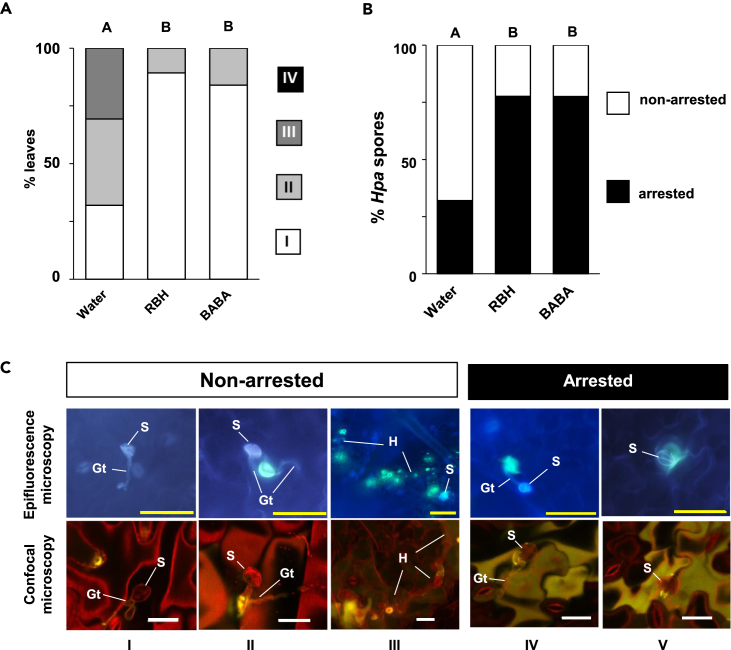
β-amino acid-induced resistance against *Hyaloperonospora arabidopsidis* (A) Colonization of Arabidopsis leaves (Col-0) by downy mildew pathogen *Hyalopoeronospora arabidopsidis* WACO9 (*Hpa*) after treatment of the soil with R-β-homoserine (RBH) and β-aminobutyric acid (BABA). Seedlings were challenged with *Hpa* conidiospores at 2 days after treatment with water, 1.5 mM RBH or 0.1 mM BABA. Resistance was quantified at 6 days post inoculation (dpi) by assigning trypan-blue stained leaves to four classes of *Hpa* colonization (Schwarzenbacher et al. 2020): healthy leaves (I), leaves showing hyphal colonization with <10 conidiophores/leaf (II), extensive hyphal colonization with ≥10 conidiophores/leaf (III), and extensive hyphal growth with conidiospores and sexual oospores (IV). Shown are relative frequency distributions between *Hpa* colonization classes. Different letters indicate statistically significant differences between treatments (Fisher’s exact tests+ Bonferonni correction; p < 0.05; n = 70–80 leaves; the experiment has been repeated once with similar results). (B) Quantification of penetration resistance in water-, RBH-, and BABA-treated plants at the stage of spore germination and early hyphal colonization (3 dpi), using epifluorescence microscopy analysis of aniline blue/calcofluor-stained leaves. Shown are percentages of callose-arrested and non-arrested conidiospores, as detailed in Figure 1C. Different letters indicate statistically significant frequency differences between treatments (Fisher’s exact tests+ Bonferonni correction; p < 0.05; n > 100 conidiospores; the experiment has been repeated once with similar results). (C) Germinated conidiospores were categorized as “non-arrested” if no callose was deposited at the germinated spore (I), or if the germ tube/expanding hyphae penetrated through the callose (II and III). Conidiospores were categorized as “arrested” if the callose encapsulated the tip of the germ tube (IV) or the adhesion site of the spore (V). Shown are representative examples by epifluorescence microscopy (upper panel) or confocal laser scanner microscopy (lower panel) after double-staining with aniline blue/calcofluor or aniline blue/direct red-23, respectively. Conidiospores, germ tubes and expanding hyphae are indicated by S, Gt, and H, respectively. Callose fluorescence is indicated by yellow/green. Yellow and white bars at the bottom of each photo represent scale bars 50 μm and 20 μm, respectively.

We have previously shown that root application of RBH or BABA primes callose-related defense in Arabidopsis at the early stages of colonization by *Hpa.*^6^^,^^23^ To compare the effects of both agents on this primed defense response, 2-week-old Arabidopsis seedlings were soil-drenched with 1.5 mM RBH and 0.1 mM BABA, which resulted in comparable levels of IR against *Hpa* by 6 days post inoculation (dpi; Figure 1A). To compare the contributions of callose-related defense to RBH- and BABA-IR, we collected leaves at relatively early time point of 3 dpi for double-staining with aniline blue (to visualize callose) and calcofluor (to visualize *Hpa*). Epifluorescence microscopy confirmed that RBH and BABA increased the proportion of *Hpa* spores elicited callose depositions that prevent spores germination and/or early hyphal colonization. This enhanced penetration resistance was statistically significant in comparison to leaves from un-primed control plants, which displayed higher numbers of germinating *Hpa* spores that penetrated the callose and advanced into the tissue (Figures 1B and 1C). Furthermore, quantification of callose in plants that had not been challenged with *Hpa* revealed that treatments with RBH and BABA do not increase callose directly (Figures S1A and S1B), confirming that these chemicals act as priming agents of this defense layer.

**Figure 2:**
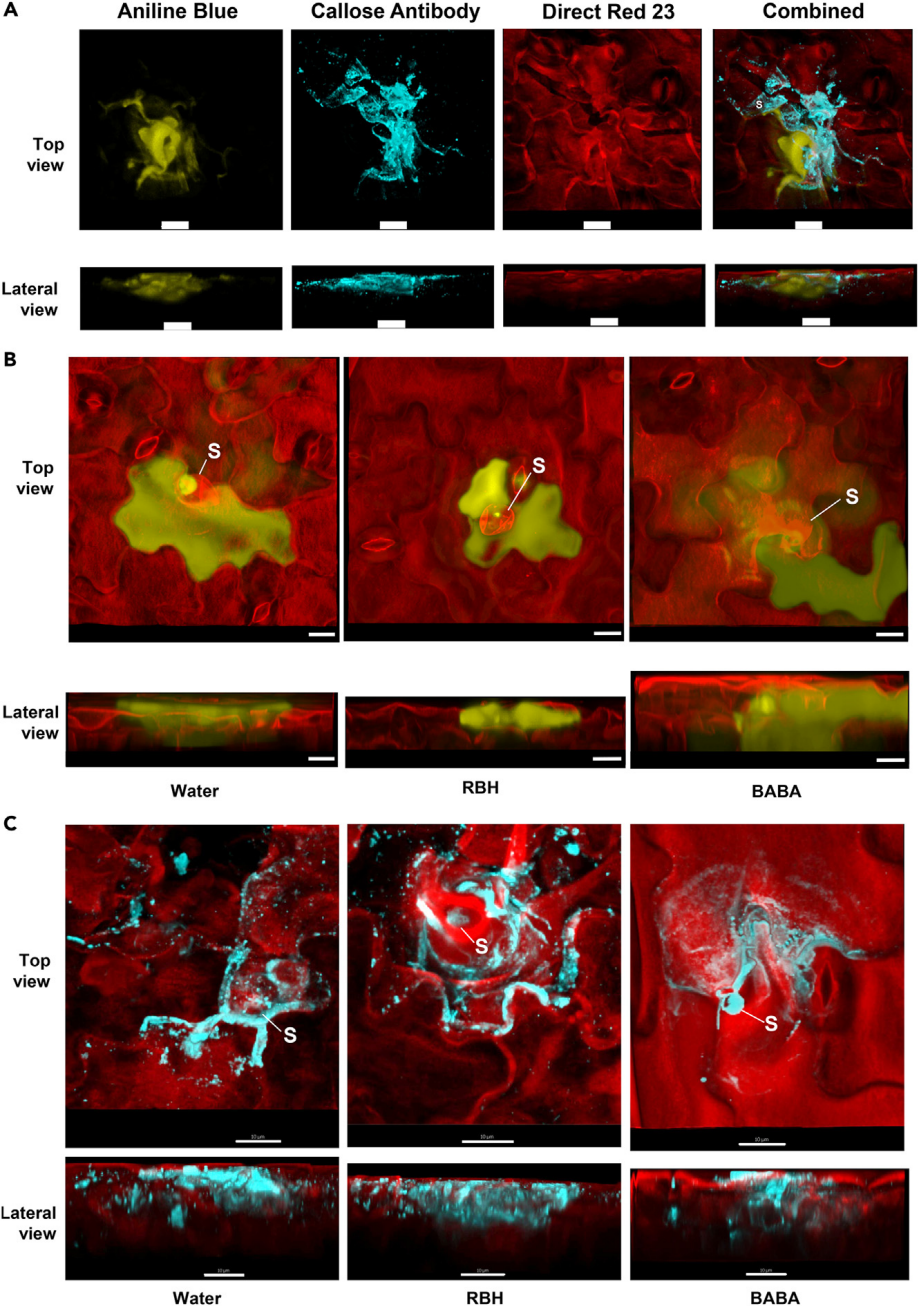
*Hpa*-arresting callose papillae in water-, RBH-, or BABA-treated plants (A) Top and lateral views of a representative *Hpa*-arresting papilla after triple staining by aniline blue, a monoclonal antibody against the β-1,3 glucan of callose, and Direct Red 23 at 3 dpi. Shown are 3-dimensional image reconstructions of z-stacked scans by Morphographx software. White scale bars correspond to10 μm. (B and C) Top and lateral views of (B) aniline blue (yellow) and direct red-23 (red) or (C) β-1,3 glucan antibody (blue) and direct red-23 (red) stained *Hpa*-arresting callose papillae in water-, RBH-, or BABA-treated plants at 3 dpi after 3-dimensional image reconstruction of z-stacked scans using Morphographx software. Conidiospores are indicated by S; white scale bars correspond to 10 μm.

To obtain a better impression of the structure of these *Hpa*-arresting papillae, leaves were analyzed by confocal scanning laser microscopy after double-staining with aniline blue (to visualize callose) and direct red-23 (to visualize the cell walls of *Hpa* and the plant cell). Unexpectedly, the aniline blue-stained depositions associating with the more excessive structures around arrested *Hpa* spores (classes IV and V) appeared more diffuse and widespread than the discrete depositions trailing the expanding hyphae of non-arrested *Hpa* (classes II and III). To test whether this diffuse aniline blue signal is associated with callose, we employed immunostaining with a monoclonal antibody against the β-1,3-glucan of callose.^24^ While triple staining by aniline blue, antibodies, and direct red-23 revealed substantial co-localization, the fluorescence signal from the aniline blue staining appeared more diffuse than the discrete fluorescence signal from the secondary antibody (Figure 2A). Since aniline blue staining is not chemically specific to callose and can also detect α-1,4-glucan, glycoproteins, and proteoglycans,^25^^,^^26^ these results suggest that the wider and more protracted aniline blue signals associated with *Hpa*-arresting papillae involve more than callose only. To search for potential differences between these *Hpa*-arresting structures in control-, RBH-, and BABA-treated plants, we reconstructed three-dimensional models from z-stacked confocal microscopy scans of *Hpa*-arresting papillae upon staining by either aniline blue, or β-1,3-glucan antibody (Figures 2B and 2C, respectively). Apart from a difference in the frequency of these *Hpa*-arresting papillae, both analyses failed to detect obvious differences in the morphology of the papillae between water-, RBH- and BABA-treated plants.

### Role of PMR4 in RBH-and BABA-IR against *Hpa*

**Figure 3:**
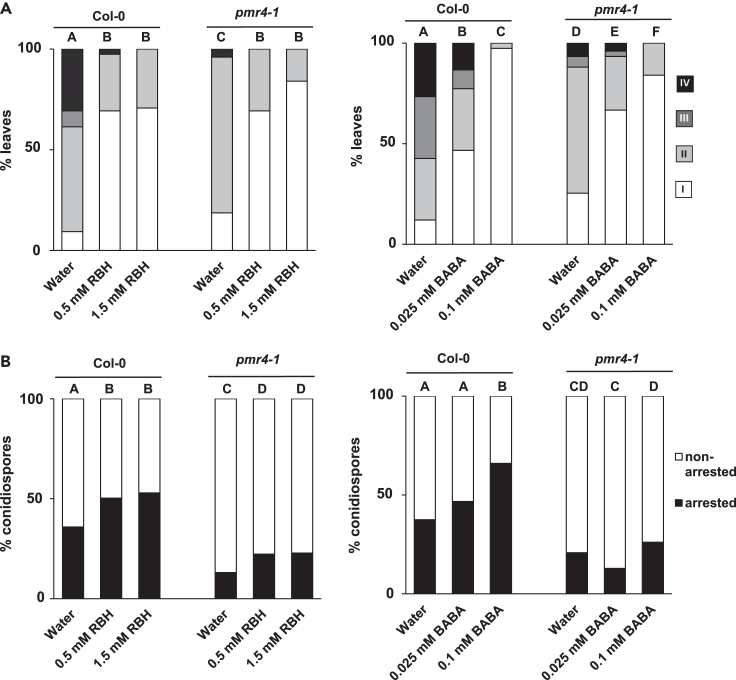
Role of the callose synthase PMR4 in RBH- and BABA-induced penetration resistance (A) *Hpa* colonization in leaves of Col-0 and *pmr4-1* at 6 dpi after pre-treatment with water (control) or increasing concentrations of RBH or BABA. For details, see the legend of Figure 1A. For each priming agent, different letters indicate statistically significant differences between genotype-concentration combinations (Fisher’s exact tests+ Bonferonni correction; p < 0.05; n = 70–80 leaves; the experiment has been repeated once with similar results). (B) Penetration resistance by callose papillae in leaves of Col-0 and *pmr4-1* seedlings at 3 dpi after pre-treatment with water (control) or increasing concentrations of RBH or BABA. For details, see legends of Figures 1B and 1C. For each priming agent, different letters indicate statistically significant frequency differences between treatment-concentration combinations (Fisher’s exact tests+ Bonferonni correction; p < 0.05; n > 100 conidiospores; the experiment has been repeated once with similar results).

Although RBH- and BABA-IR against *Hpa* are tightly associated with increased deposition of pathogen-arresting callose papillae, the contribution of the pathogen-responsive callose synthase PMR4 in these IR responses has never been investigated. To address this question, the degree of *Hpa* leaf colonization was compared at 6 dpi between wild type (Col-0) and *pmr4-1* plants following pre-treatment of the roots with water (control) or increasing concentrations of RBH or BABA. Water-treated *pmr4-1* showed enhanced basal resistance compared to water-treated Col-0, supporting previous reports that the *pmr4-1* mutation increases SA-dependent resistance to biotrophic pathogens (Figure 3A).^13^^,^^27^ Secondly, all RBH and BABA concentrations statistically reduced *Hpa* colonization compared to the water-treated controls, which was apparent in both Col-0 and *pmr4-1* (Figure 3A). Hence, PMR4 is not critical for either IR response. Nonetheless, despite the elevated basal resistance of *pmr4-1*, this mutant still displayed hyphal colonization by *Hpa* and failed to reach the near-complete level of BABA-IR observed in wild-type plants after pre-treatment with 0.1 mM BABA. These results indicate that PMR4 has a quantitative contribution to BABA-IR against *Hpa* (Figure 3A).

### Role of PMR4 in RBH- and BABA-induced penetration resistance

For both RBH-IR and BABA-IR, the degree of non-sporulating *Hpa* at 6 dpi (trypan-blue staining classes I and class II; Figure 1A) is higher than the degree of early *Hpa* arrestment at 3 dpi (aniline blue staining classes IV and V; Figure 1B), supporting the notion that IR is a quantitative immune response that involves a multitude of early and late-acting defense layers.^1^^,^^28^ Accordingly, it is possible that the contribution of PMR4-dependent callose to IR at 6 dpi is masked by other defense layers that act after early penetration resistance. To test this hypothesis, the frequency of *Hpa*-arresting callose papillae was compared between water, RBH-, and BABA-treated Col-0 and *pmr4-1* at the relatively early time point of 3 dpi, when the resistance to *Hpa* coincides with depositions of callose-rich papillae (Figures 1B and 1C). Although the intensity and efficiency of *Hpa*-arresting papillae were dramatically reduced in *pmr4-1* compared to Col-0, there was still a residual level of *Hpa*-induced callose in *pmr4-1* (Figures 3B and S2), which supports earlier reports of *PMR4*-independent callose deposition.^15^^,^^22^^,^^29^ Interestingly, while BABA failed to enhance penetration resistance in *pmr4-1*, RBH still enhanced the frequency of *Hpa*-arresting callose papillae in *pmr4-1* (Figure 3B). Hence, RBH- and BABA-induced penetration resistance differs in their requirement for PMR4.

### Role of PDLP proteins in RBH- and BABA-induced penetration resistance

**Figure 4:**
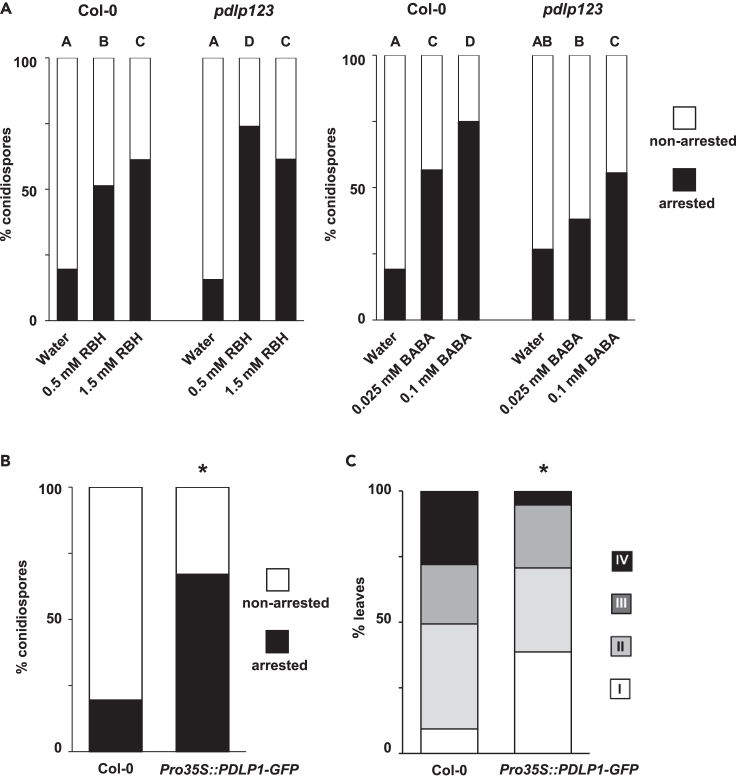
Role of PDLPs in RBH- and BABA-induced penetration resistance against *Hpa* (A) Penetration resistance by callose papillae in leaves of Col-0 and *pdlp123* at 3 dpi after pre-treatment of the soil with water (control) or increasing concentrations of RBH or BABA. For details, see the legend of Figures 1B and 1C. For each priming agent, different letters indicate statistically significant frequency differences between treatment-concentration combinations (Fisher’s exact tests+ Bonferonni correction; p < 0.05; n > 100 conidiospores; the experiment has been repeated once with similar results). (B) Penetration resistance by callose papillae in leaves of Col-0 and *Pro35S*::*PDLP1-GFP* at 3 dpi. For details, see legends of Figures 1B and 1C. Control samples (Col-0) in (B) are shared with control samples (water-treated Col-0) of RBH-treated plants in (A). Asterisk indicates statistically significant frequency differences from Col-0 (Fisher’s exact tests+ Bonferonni correction; p < 0.05; n > 100 conidiospores; the experiment has been repeated once with similar results). (C) *Hpa* colonization at 6 dpi in leaves of Col-0 and *Pro35S*::*PDLP1-GFP*. For details, see the legend of Figure 1A. Asterisk indicates statistically significant frequency differences from Col-0 (Fisher’s exact tests+ Bonferonni correction; p < 0.05; n = 70–80 leaves; the experiment has been repeated once with similar results).

Caillaud et al. (2014) reported that the PDLP triple mutant *pdlp123* is compromised in callose deposition around *Hpa* haustoria and that PDLP1 locates to extrahaustorial membrane before callose formation.^22^ These results suggested that PDLP proteins guide callose formation to prevent functional haustoria at the relatively late stages of the Arabidopsis-*Hpa* interaction. To investigate whether PDLPs also contribute to RBH- and BABA-induced penetration resistance at earlier stages of the interaction, we quantified *Hpa*-arresting callose papillae in Col-0 and *pdlp123* plants after pre-treatment with water and increasing concentrations of RBH or BABA. The BABA-induced penetration resistance was abolished in the *pdlp123* mutant after treatment with 0.025 mM BABA and strongly attenuated after treatment with 0.1 mM BABA (Figure 4A). By contrast, RBH-induced penetration resistance was unaffected in *pdlp123* plants compared to wild-type plants and even appeared more pronounced after treatment with the relatively low dose of 0.5 mM RBH (Figure 4A). Hence, PDLP proteins specifically contribute induced penetration resistance by BABA and not by RBH, reinforcing our notion that RBH- and BABA-induced penetration resistance to *Hpa* are controlled by different pathways. To study further the contribution of PDLP1 to penetration resistance, we quantified *Hpa*-arresting callose papillae and *Hpa* colonization in *35SPro*::*PDLP1-GFP* over-expression plants and Col-0. The *35SPro*::*PDLP1-GFP* line showed strongly increased levels of callose-associated penetration resistance at 3 dpi (Figure 4B), which was associated with reduced *Hpa* colonization by 6 dpi (Figure 4C). Hence, increased expression of *PDLP1* mimics chemical priming of callose-mediated penetration resistance against *Hpa*.

### BABA primes early translocation of PDLP1 to germinating *Hpa* spores

**Figure 5:**
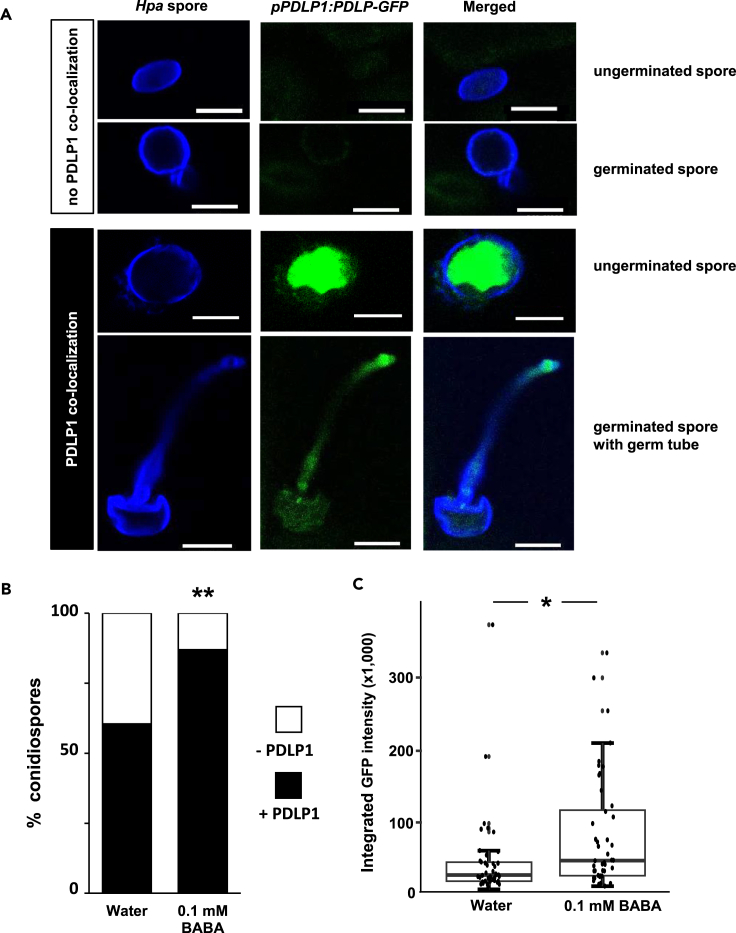
BABA primes early translocation of PDLP1 to germinating *Hpa* conidiospores (A) Representative examples of PDLP1-GFP in leaves of *ProPDLP1*::*PDLP-GFP* plants relative to *Hpa* conidiospores at 1 dpi. Leaves were stained with calcofluor and imaged by confocal microscopy in the same focal plane. Calcofluor-stained spores and germ tubes are shown in blue; PDLP1-GFP is shown in green. White bars represent scale bars (10 μm). (B) Co-localization of PDLP1-GFP with *Hpa* conidiospores in leaves of water- and BABA-treated *ProPDLP1*::*PDLP-GFP* plants. Shown are frequency distributions of PDLP1-GFP that either co-localize (+PDLP1), or do not co-localize (-PDLP1) with *Hpa* conidiospores at 1 dpi, as illustrated in (A). Asterisks indicate a statistically significant difference to the water treatment (water; Fisher’s exact test, ∗∗:p < 0.01, n = 40–50 conidiospores; the experiment has been repeated once with similar results). (C) Quantification of PDLP1-GPP signal intensity at *Hpa* spores. Shown are integrated fluorescence intensities of GFP that co-localize with *Hpa* spores. Boxplots show median (middle bar), interquartile range (IQR; box), 1.5 x IQR (whiskers) and replication units (single dots) of integrated fluorescence intensities of GFP. Asterisk indicates a statistically significant difference in the *Hpa* co-localizing GFP signal between water- and BABA-treated plants (Welch t test, ∗:p < 0.05, n = 40–50 spores; the experiment has been repeated once with similar results).

To confirm that PDLP1 drives BABA-induced penetration resistance to *Hpa*, we stained leaves with calcofluor white and used laser scanning confocal microscopy to study the subcellular localization of PDLP1 relative to *Hpa* in water- and BABA-treated plants carrying the translational reporter construct *ProPDLP1*::*PDLP1-GFP*. This analysis was performed at 1 dpi, in order to capture the early signaling events preceding the deposition of augmented callose deposition in primed plants (2–3 dpi) and was based on the same focal plane to ascertain co-localization between PDLP1-GFP and *Hpa*. Pre-treatment with 0.1 mM BABA not only increased the co-localization of *Hpa* spores with PDLP1-GFP (Figure 5B), but it also intensified the PLDP1-GFP signal at *Hpa* spores (Figure 5C). Hence, BABA primes co-localization of PDLP1 with germinating *Hpa* spores during the onset of induced penetration resistance (Figures 5A–5C and S3). Interestingly, no increased co-localization of PDLP1-GFP with *Hpa* spores was observed in RBH-primed plants following *Hpa* inoculation (Figure S4), strengthening our conclusion that RBH- and BABA-induced penetration resistance are regulated via different pathways.

## Discussion

### Penetration resistance against *Hpa*: A multifaceted immune response

RBH and BABA can induce near complete levels of resistance against *Hpa* in susceptible Arabidopsis genotypes (Figure 1A).^6^^,^^23^^,^^30^ Although both β-amino acids share the same cellular transporter,^9^ they are not perceived by the same receptor, act at different concentrations, and differ in phytotoxicity at higher doses.^6^^,^^9^ Despite these differences, both agents induce high levels of penetration resistance to *Hpa*, which is based on a priming of callose-associated penetration defense at germinating conidiospores and expanding germ tubes. While these *Hpa*-arresting papillae appeared similar in structural morphology (Figures 2B and 2C), our study demonstrates that they are controlled by different pathways (Figures 3, 4, and 5). Buswell et al. (2018) reported that RBH-IR against *Hpa* is associated with augmented induction of the tryptophan-derived phytoalexin camalexin and that the camalexin-deficient *pad3-1* mutant shows attenuated levels of RBH-IR to *Hpa*.^6^ Interestingly, previous studies have demonstrated signaling roles for tryptophan-derived secondary metabolites in penetration resistance against filamentous pathogens, such as PEN2/GSTU13-dependent products of 4-methoxy-indole-3-ylmethylglucosinolate (4MI3G), indole-3-carboxylic acid (I3CA), benzoxazinoids (BXs), and camalexin accordingly.^31^^,^^32^^,^^33^^,^^34^ It is tempting to speculate that this diversity in tryptophan-derived metabolites plays a role in the differential regulation of BABA- and RBH-induced penetration resistance against *Hpa.* Finally, it is worth noting that abscisic acid (ABA) has been implicated in the regulation of both basal resistance and BABA-IR to *Hpa.*^7^^,^^23^^,^^35^ Since PDLP1 has been shown to control ABA-induced bud dormancy in Aspen trees,^36^ it is plausible that ABA also plays a role in the priming of PDLP1-dependent penetration defense by BABA.

### The contribution of PMR4 to penetration resistance

Previous experiments with the chemical callose synthesis inhibitor 2-deoxy-D-glucose (2-DDG) have suggested a critical role for callose in IR.^37^^,^^38^^,^^39^ However, despite the dominant role of the callose synthase PMR4 in pathogen-induced immune responses, the *pmr4-1* loss-of-function mutation had no effect on RBH-IR against *Hpa* and only weakly affected BABA-IR against *Hpa* colonization by 6 dpi (Figure 3A). The latter finding suggests a quantitative contribution of PMR4 to BABA-IR against *Hpa*, which supports previous findings that BABA-IR against this pathogen involves priming of multiple SA-dependent and -independent layers of induced defense.^7^^,^^23^ The *pmr4-1* mutation showed a significantly reduced intensity of *Hpa*-induced callose deposition and compromised penetration resistance (Figure 3B), confirming its dominant role in pathogen-induced callose. However, there was still a residual level of *Hpa*-induced callose in the *prm4-1* mutant (Figures 3B and S2), which supports previous observations by Dong et al. (2008) and Caillaud et al. (2014).^22^^,^^29^ Interestingly, while RBH still increased the frequency of *Hpa*-arresting callose papillae in *pmr4-1*, BABA failed to do so (Figure 3B). Hence, BABA-induced penetration resistance to *Hpa* fully depends on PMR4, whereas RBH-induced penetration resistance involves priming of additional callose synthases. Other callose synthases have been implicated in pathogen-induced callose. For instance, defense-related callose has been observed in the *pmr4-1* mutant upon treatment with chitosan and during infection by *Hpa.*^22^^,^^29^ Dong et al. (2008) showed that besides *PMR4* (*CalS12*), the expression of the *CalS1* is also induced by both SA and *Hpa* in an NPR1-dependent manner.^29^ Furthermore, both CalS1 and CalS8 have been implicated in ROS-dependent callose deposition at plasmodesmata.^40^ It is therefore plausible that CalS1 and/or CalS8 have a contribution to RBH-induced penetration resistance against *Hpa*.

### The contribution of PDLPs to penetration resistance

While PDLPs have mainly been implicated in the regulation of callose deposition at plasmodesmata,^19^^,^^20^^,^^21^ PDLP1 has also been reported to co-localize with *Hpa* haustoria and mediate PMR4-dependent callose deposition during the relatively advanced stages of the interaction.^22^ Here, we have shown that the *pdlp123* mutant is strongly attenuated in BABA-induced penetration resistance against *Hpa* (Figure 4A) and that genetic over-expression of *PDLP1* mimics BABA-induced priming of early acting penetration defense (Figures 4B and 4C). Together with our finding that BABA primes the co-localization of PDLP1 at *Hpa* spores during the onset of BABA-induced penetration resistance (Figures 5A–5C), we propose that PDLP1 regulates the augmented induction of PMR4-dependent penetration resistance by BABA. Interestingly, PDLP1 and PDLP5 have recently been shown to be part of a protein complex with the immune regulatory protein NDR1/HIN1-LIKE 3 (NHL3), which mediates CalS1-dependent callose and defense-related plasmodesmatal closure.^41^ However, PDLP5, unlike PDLP1, did not co-localize with *Hpa* haustoria, while NHL3 was not identified as a direct interactor with PDLP1, suggesting that PDLP1 acts through a different signaling complex to regulate *Hpa*-induced callose.^22^ Our finding that BABA primes PDLP1 localization at the onset of PMR4-dependent penetration resistance provides further evidence for a specific defense regulatory role of this protein in the Arabidopsis-*Hpa* interaction.

### Limitations of the study

Although our study has revealed that PDLPs and PMR4 are key regulators of BABA-induced penetration resistance against *Hpa*, the machinery driving RBH-induced penetration resistance remains largely unknown. For instance, the callose synthetase responsible for the increased deposition of *Hpa*-arresting papillae in RBH-primed *pmr4-1* plants remains to be identified. Furthermore, the cellular mechanisms by which PDLPs are mobilized to germinating *Hpa* spores in BABA-primed plants and orchestrate the onset of PMR4-dependent callose deposition await further investigation.

## STAR★Methods

### Key resources table

**Table:** 

REAGENT or RESOURCE	SOURCE	IDENTIFIER
**Antibodies**		

(1-3)-beta-glucan antibody	Biosupplies	Cat#400-2; RRID: AB_2747399
Goat anti-Mouse IgG conjugated with Alexa Fluor™ 488	Invitrogen	Cat#A11001; RRID: AB_2534069

**Chemicals, peptides, and recombinant proteins**		

BABA	Sigma-Aldrich	Cat#A44207
RBH	Ark Pharm	Cat#AK-23884
Aniline blue	Sigma-Aldrich	Cat#4415049
Calcofluor white	Sigma-Aldrich	Cat#F3543
Direct red-23	Scientific Laboratory Supplies	Cat#CHE1866
Trypan Blue	Sigma-Aldrich	Cat#T6146
Chloral hydrate	Sigma-Aldrich	Cat#23100
Phenol	Fisher Scientific	Cat#BP226-100
Glycerol	Fisher Scientific	Cat#G/0650/17
DL-Lactic acid, 90%	Fisher Scientific	Cat#10031120

**Experimental models: Organisms/strains**		

*Arabidopsis thaliana:*wild type Col-0	NASC	N/A
*Arabidopsis thaliana:* mutant *pmr4-1*	Nishimura et al.^13^	N/A
*Arabidopsis thaliana: mutant pdlp123*	Thomas et al.^20^	N/A
*Arabidopsis thaliana:35SPro::PDLP1-GFP* in Col-0	Thomas et al.^20^	N/A
*Arabidopsis thaliana:PDLP1Pro::PDLP1-GFP* in Col-0	Thomas et al.^20^	N/A

**Deposited data**		

[Mendeley Data]: https://doi.org/10.17632/mrnz8ck22c.1		

**Software and algorithms**		

ImageJ (Fiji)	Rueden et al.^42^	https://fiji.sc/
MorphoGraphX (version 2.0)	Strauss et al.^43^	https://www.morphographx.org/
R v 4.0.3	R	http://R-project.org
R Studio 2023.12.0	RStudio, Inc	https://rstudio.com
R:fifer	N/A	https://fifer_1.1.tar.gz

### Resource availability

#### Lead contact

Requests for resources, information and datasets should be directed to and will be fulfilled by the Lead Contact, Jurriaan Ton (j.ton@sheffield.ac.uk).

#### Materials availability

This study did not generate new unique reagents or germplasm.

##### Data and code availability

Data: All the raw data generated during this study are available at “Mendeley Data: https://doi.org/10.17632/mrnz8ck22c.1.” All raw microscopy files are available from the lead contact upon request.

Code: This study did not generate any original code.

Any other items: Any additional information required to reanalyse the data reported in this paper is available from the lead contact upon request.

### Experimental models and study participant details

#### Biological material and cultivation

All *Arabidopsis thaliana* lines used in this research are in the genetic background of the Columbia (Col-0) accession. The *pmr4-1* mutant was identified and characterised by Nishimura et al. (2003), while the *pdlp123*, *35Spro::PDLP1-GFP* and *ProPDLP1::PDLP-GFP* lines have been described and characterised by Thomas et al. (2008) and Caillaud et al. (2014).^20^^,^^22^ Arabidopsis seeds were suspended in 0.18% agar, stratified for 2-3 days in the dark at 4°C, and sowed onto 2:1 (v/v) Scott’s Levington M3 compost/sand mixture. Plants (∼20-25 per 70-mL pot) were then cultivated in a climate chamber (Conviron, PGC Flex) containing Valoya NS1 LED lights at 150 μmol/m^2^/s, using a 8.5h light (21°C)/15.5h dark (18°C) cycle at ∼60% relative humidity. *Hyaloperonospora arabidopsidis* (*Hpa*) strain WACO9 was kept in its asexual cycle by alternately inoculated Col-0 and WsNahG with *Hpa* conidiospores.

### Method details

#### Induced resistance bioassays

Two-week-old seedlings were soil-drenched with water (control), BABA (Sigma-Aldrich, #A44207) or RBH (Ark Pharm, #AK-23884) by injecting a 10x concentrated solution at 10% of the pot volume. Two days after chemical treatment, plants were spray-inoculated with *Hpa* conidiospores (10^5^ spores/mL) and kept in sealed containers at 100% RH to promote infection.

For quantification of callose-associated penetration resistance, seedlings were harvested at 3 dpi and cleared overnight (ON) in 100% ethanol. Samples were washed twice in dH2O for 15 min and double-stained with a 4:1mixture of 0.05% (w/v) aniline blue (Sigma-Aldrich, #4415049) in 0.07M phosphate buffer and 0.005% (w/v) calcofluor white (Sigma-Aldrich, #F3543) in 0.1M Tris-HCl (pH=8.5) for 15 minutes. After the double staining, samples were transferred to 0.05% (w/v) aniline blue (Sigma-Aldrich, #4415049) in 0.07M phosphate buffer and incubated overnight (ON). All the staining steps were performed at room temperature (RT) in the dark. Two true leaves from each plant and five plants per genotype/treatment combination were analysed by epifluorescence microscopy (Leica DM6B; light source: CoolLED pE-2; 365 nm excitation filter, L 425 nm emission filter, 400 nm dichroic filter). Germinated conidiospores on the ten leaves were categorised into ‘arrested’ (spore adhesion site or tip of germination tubes fully encased in callose) or ‘non-arrested’ (no callose or callose deposited alongside germination tubes/hyphae but not encased the tip of germination tubes) as further detailed and illustrated in Figure 1C.

For quantification of *Hpa* colonisation, shoot material was harvested at 6-7 dpi and stained with lactophenol trypan blue solution (10 mg trypan blue, 5g phenol, 5g glycerol, 5 ml lactic acid, 10 ml distilled water, 30 ml 100% ethanol) to quantify total IR.^44^ Samples were kept in 15 ml centrifuge tubes with lactophenol trypan blue solution and boiled for 1 min. Tubes were opened immediately after boiling to release the pressure for improving the infiltration of the staining solution. Samples were rested in RT for 5 mins, followed by another 1 min bioling, then stayed in RT for 3 hours. After the staining process, seedlings were transferred to 3.63 M chloral hydrate solution to destain ON. *Hpa* colonisation was quantified by categorising leaves from 15-20 plants into four distinct classes (I-IV), ranking from no hyphal colonisation (I), hyphal colonisation with 0 to 9 conidiospores (II), hyphal colonization with 10 or more conidiophores (III) to major hyphal colonisation with deposition of conidiospores and oospores (IV).^7^

To quantify the direct effects of RBH and BABA on callose before *Hpa* challenge, plants were treated as described above, harvested 2 days after chemical treatment, and cleared ON in 100% ethanol. Samples were washed twice in dH_2_O for 15 min. and stained ON with 0.05% (w/v) aniline blue (Sigma-Aldrich, #4415049) in 0.07M phosphate buffer. Two true leaves from each plant and six plants of each genotype/treatment combination were anlaysed by epifluorescence microscopy (Leica DM6B; light source: CoolLED pE-2; 365 nm excitation filter, L 425 nm emission filter, 400 nm dichroic filter). Two sites from each leaf were imaged for callose quantification. Callose was quantified in each image by using analyze particles function in Fiji/ImageJ software.^42^^,^^45^

#### Confocal laser scanning microscopy imaging of callose papillae

Three days after *Hpa* inoculation, seedlings were collected and destained ON in 100% ethanol to remove the chlorophyll. Samples were then washed twice in dH_2_O for 15 mins and stained ON with 1:1 mixture of 0.05% (w/v) aniline blue (Sigma-Aldrich, #4415049) in 0.07M phosphate buffer and 0.2% direct red-23 (Scientific Laboratory Supplies, #CHE1866) at RT under dark condition. For triple staining with aniline blue, the (1-3)-beta-glucan antibody (Biosupplies, #400-2) and direct red-23, the primary antibody was applied after ON staining with aniline blue and direct red-23. All the steps were carried out in the dark condition at RT. Samples were removed from the aniline blue/direct red-23 mixture and washed twice in PBS buffer (pH 7.4) for 10 min., after which leaf samples were transferred into the 3% non-fat milk (w/v) prepared with pH 7.4 PBS for 30 min. blocking, followed by two wash steps of 10 min. in PBS buffer (pH 7.4). Samples were then transferred into blocking solution containing 50x diluted primary antibody for 90 min. after which the samples were washed three times in PBS buffer (pH 7.4). Goat anti-Mouse IgG conjugated with Alexa Fluor™ 488 (Invitrogen, #A11001) was used as the secondary antibody, which was diluted 100 times in blocking solution before use. Samples were incubated for 60 min in the secondary antibody solution and washed three times (5 min. each) with PBS buffer (pH 7.4).

For double staining with direct red-23 and the (1-3)-beta-glucan antibody, leaves were washed twice in dH_2_O for 15 min and stained with 0.1% direct red-23 ON at RT in the dark, followed by the same antibody staining procedure as described above. Leaves were mounted in water and imaged with a Nikon A1 confocal scanning microscope, using Plan Fluor 40× oil immersion object lens (numerical aperture=1.3). Aniline blue was excited at 405 nm and emission was collected at 425-475 nm; goat anti-Mouse IgG conjugated with Alexa Fluor™ 488 was excited at 488 nm and emission was collected at 500-550 nm; direct red 23 was excited at 562 nm and emission was collected at 570-620 nm. Z-steps was set to 1μm for the triple staining with aniline blue, the (1-3)-beta-glucan (callose) antibody and direct red-23, 0.4 μm for the double staining with aniline blue and direct red-23, and 0.5 μm for the double staining with the (1-3)-beta-glucan antibody and direct red-23. Reconstruction of the z-stacked images was performed by MorphoGraphX software.^46^

#### Co-localisation between PDLP1-GFP and *Hpa* conidiospores

One day after *Hpa* inoculation, *ProPDLP1::PDLP-GFP* seedlings were collected and vacuum-infiltrated with calcofluor white (1.25μg/mL; Sigma-Aldrich, #F3543) to stain *Hpa* conidiospores and germination tubes. Two true leaves from each plant and three plants of each treatment were imaged. Each leaf was imaged four to six sites with one to three spores on each site. Co-localisation between spores and PDLP1-GFP was analysed and imaged by confocal scanning microscopy (Nikon A1) using a Plan Fluor 40× oil immersion object lens (numerical aperture=1.3). Calcofluor white was excited at 405 nm and emission was collected at 425-475 nm; GFP was excited at 488 nm and emission was collected at 500-550 nm. With an optical section thickness of 890 nm, scanned sections included both the spore located on the leaf surface and the epidermal cell wall below the cuticle layer, considering that the Arabidopsis cuticle thickness is less than 100 nm.^47^ Z-steps was set to 0.5 μm. MorphoGraphX was used to reconstruct 3D models from z-stacked images. Integrated fluorescence intensity was quantified by Fiji/ImageJ software.^42^ Statistically significant differences in PDLP-GFP co-localisation and intensity were assessed with R software (v 4.0.3), using a Fisher’s exact test (package ‘fifer’; fifer_1.1.tar.gz) and a Welch t-test, respectively.

### Quantification and statistical analysis

All the experiments have been repeated once with similar results. Detailed information about sample number and statistical tests can be found in figure legends and the ‘Method details’ section. All statistical tests were performed with R software (v 4.0.3). Statistical differences in the frequency of callose-arrested *Hpa* spores, the frequency of leaves across *Hpa* colonisation classes, and the frequency of co-localisation between *Hpa* spores and PDLP1-GFP, were based on pairwise Fisher’s exact tests with a Bonferroni false-discovery rate correction, using R software (v 4.0.3), package ‘fifer’ (fifer_1.1.tar.gz). Statistical significance of quantitative differences in PDLP1-GFP intensity was determined with a Welch t-test, using the t.test () function in base R; quantitative differences in callose intensity after chemical treatments were tested by one-way ANOVA of log_10_-transformed data, using the aov() function in base R. Data normality and homogeneity of variances were verified by normal probability plots and residuals versus fits plots.
