# Supplementary material for: Role of PMR4 and PDLP1 in priming of early acting penetration defense by resistance-inducing β-amino acids
Source: iScience. 2024 Feb 20;27(3):109299. doi: 10.1016/j.isci.2024.109299 (PMC10933464; doi:10.1016/j.isci.2024.109299)
Supplement: Document S1. Figures S1–S4 [file mmc1.pdf]

iScience, Volume *27*

Supplemental information

Role of PMR4 and PDLP1 in priming of early acting penetration defense by resistance-inducing β-amino acids

Chia-Nan Tao and Jurriaan Ton

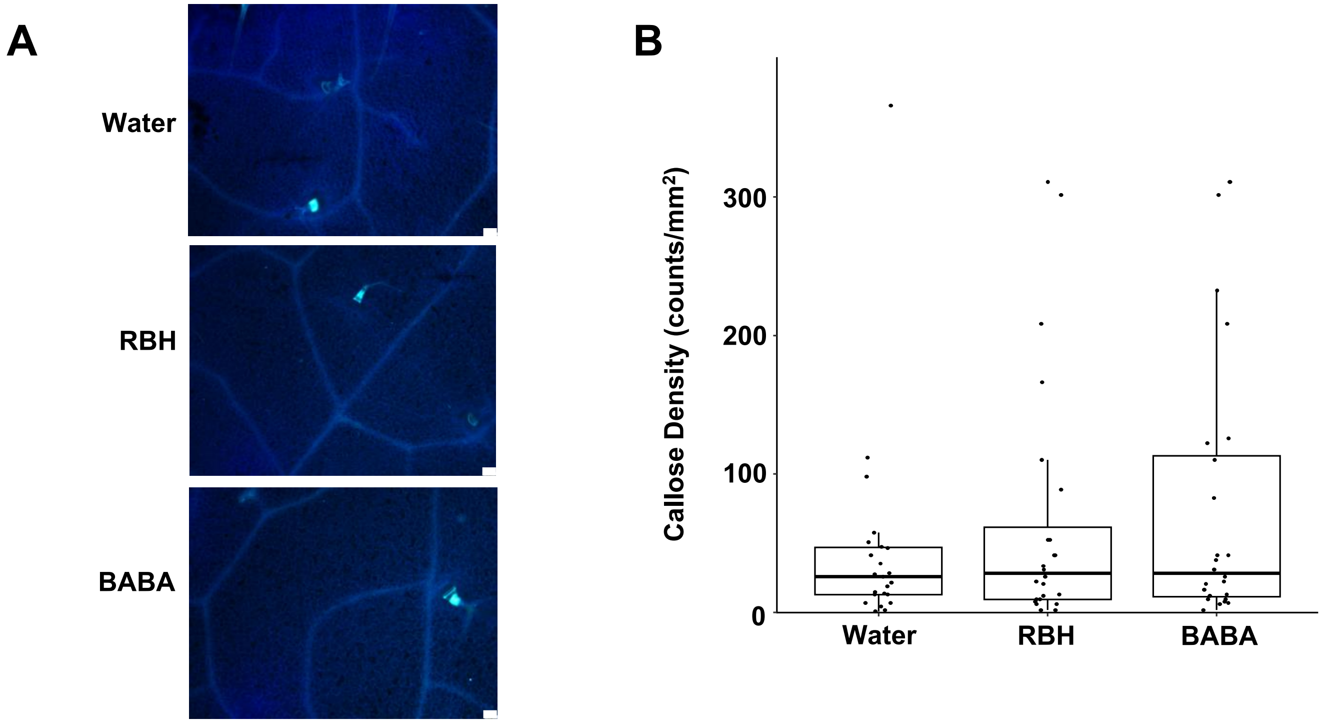

Figure S1: Callose deposition in chemically primed Arabidopsis before challenge inoculation with *Hpa*. Related to Figure 1. (A) Callose deposition was imaged by staining leaves with 0.05% aniline blue at 2 days after chemical treatment. The white bar in each photo indicates scale bar (50 µm). **(B)** Callose density (counts/mm^2) in each sample was quantified by dividing callose count from each image by total image area, as described by Jin and Mackey (2017). One-way ANOVA was performed on log-transformed density data (p=0.58; n=24 images). Results are from an experiment that was repeated once with similar results.

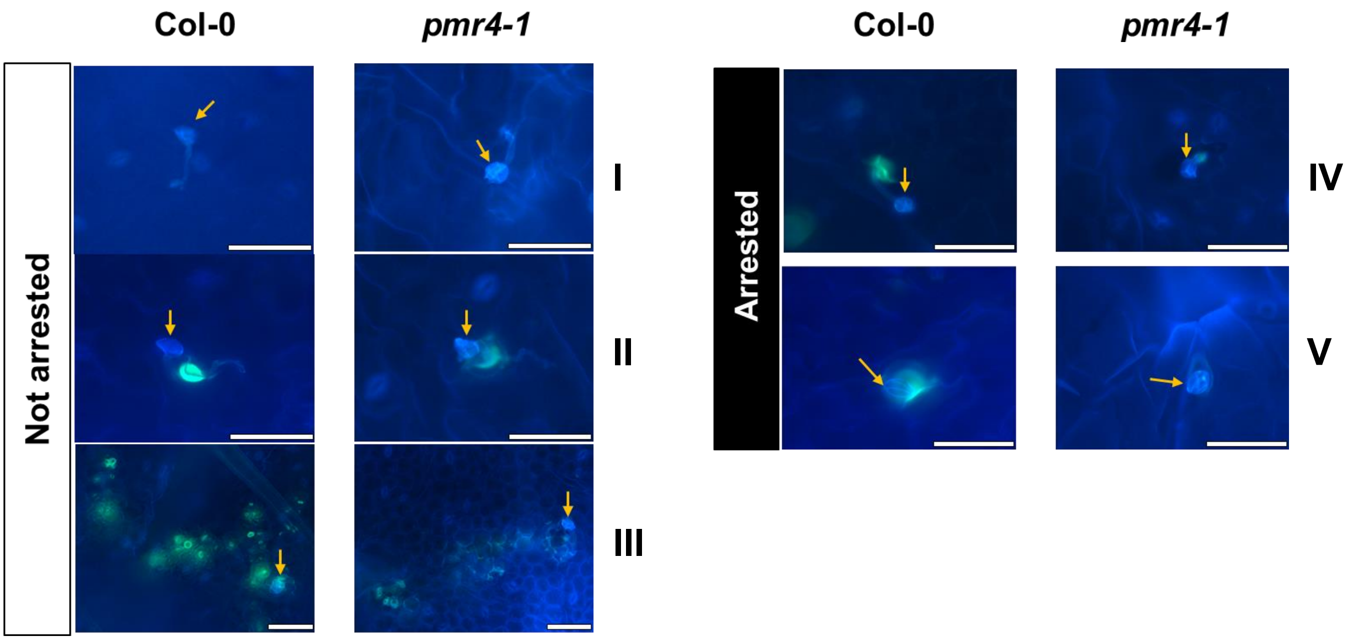

Figure S2: *Hpa*-induced callose depositions in Col-0 and *pmr4-1*. Related to Figure 2.

Shown are representative photos from epifluorescence microscopy analysis of *Hpa* conidiospores and callose papillae in aniline blue/calcofluor-stained leaves at 3 dpi. Conidiospores (orange arrows) were categorised as either ‘non-arrested’ or ‘arrested’, as detailed and illustrated in Fig. 1C. The example images for Col-0 are reused from Figure 1C. The calcofluor signal from *Hpa* is light blue; the aniline blue signal from callose depositions is yellow/green. White bars indicate scale bars (50 µm).

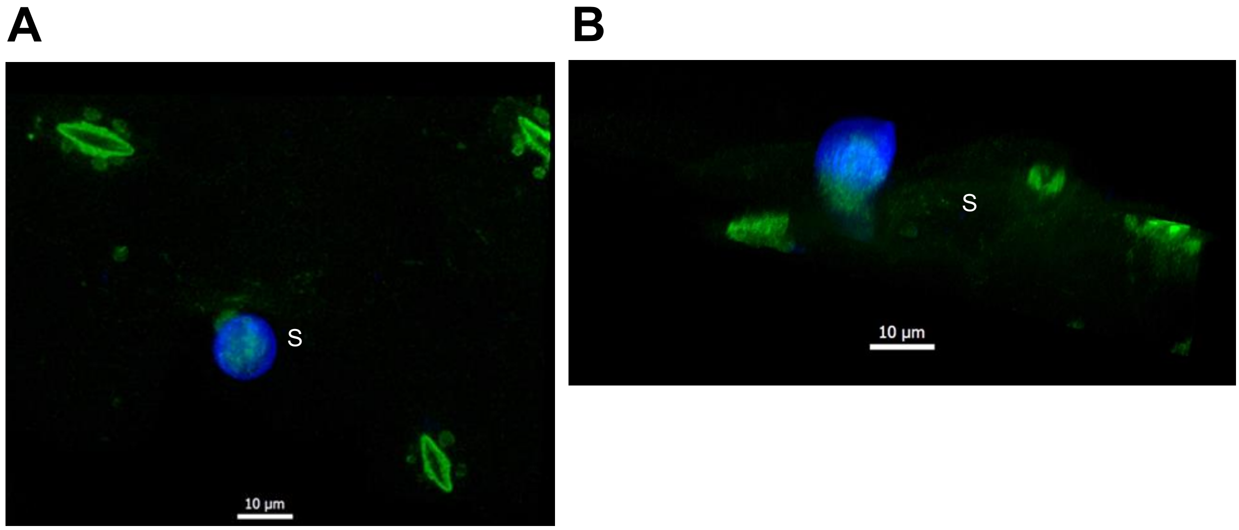

Figure S3: Co-localisation of PDLP1-GFP with *Hpa*. Related to Figure 5. Shown are top **(A)** and lateral (**B**) views of the PDLP1-GFP signal that is co-localising with a germinating conidiospore of *Hpa*. Z-stack images were taken from a calcofluor-stained *ProPDLP1::PDLP1-GFP* leaf at 1 dpi with *Hpa*. The *Hpa* spore is indicated by S. White bars represent 10 µm.

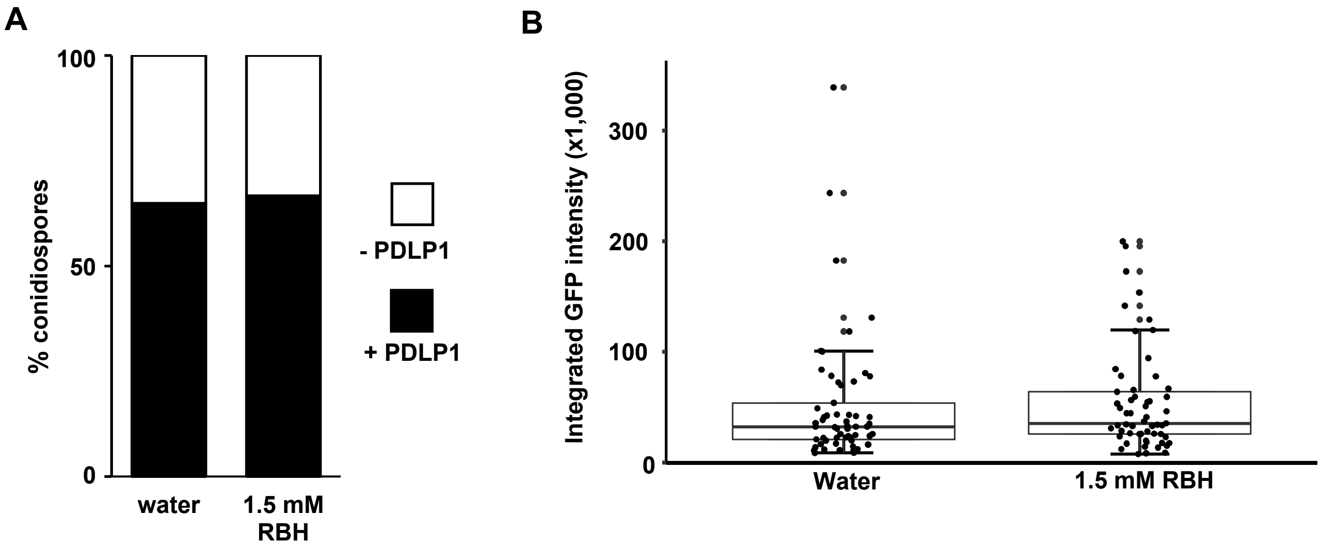

Figure S4. **RBH does not prime early translocation of PDLP1 to germinating *Hpa* conidiospores. Related to Figure 5.**

(A) Colocalization frequency between of PDLP1-GFP with *Hpa* conidiospores. Two-week-old *ProPDLP1::PDLP-GFP* seedlings were treated with water or 1.5 mM RBH and inoculated with *Hpa* 2 days later. Shown are frequency distributions of PDLP1-GFP that either co-localised (+PDLP1), or do not co-localised (-PDLP1) with *Hpa* conidiospores at 1 dpi, as illustrated in Fig. 4A (Fisher's exact test, $p>0.05$, n=57 spores; the experiment has been repeated once with similar results).

(B) PDLP1-GFP signal intensity at *Hpa* conidiospores. Shown are integrated fluorescence intensities of GFP co-localizing with *Hpa* spores. No statistically significant difference was detected in the *Hpa* co-localising GFP signal between water- and RBH-treated plants (Welch t-test, $p>0.05$, n=57 spores; the experiment has been repeated once with similar results).
